# Expression analysis and implication of Rab1A in gastrointestinal relevant tumor

**DOI:** 10.1038/s41598-019-49786-7

**Published:** 2019-09-16

**Authors:** Menglin Xu, Xinyu Shao, Xiaoyi Kuai, Liping Zhang, Chunli Zhou, Zhengwu Cheng

**Affiliations:** 1 Department of Gastrointestinal Surgery, The First Affiliated Hospital of Wannan Medical College, Wuhu, Anhui 241000 P.R. China; 2 Department of gastroenterology, The Affiliated Suzhou Hospital of Nanjing Medical University, Suzhou, Jiangsu 215006 P.R. China; 3 Department of Oncology, The First Affiliated Hospital of Wannan Medical College, Wuhu, Anhui 241000 P.R. China

**Keywords:** Colorectal cancer, Gastric cancer

## Abstract

Gastrointestinal cancers have become increasingly prevalent worldwide. Previous studies have reported an oncogenic function of Rab1A in colorectal cancer and hepatocellular carcinomas via the mTOR pathway. However, the exact role of Rab1A in gastrointestinal cancers remains elusive. We detected significantly higher expression of Rab1A in the gastrointestinal tumor tissues compared to that in other cancer types following an *in silico* analysis of TGCA and GTEX databases. Furthermore, Rab1A was overexpressed in the gastrointestinal tumor tissues compared to the para-tumor tissues. Although Rab1A expression levels were not associated with the tumor-lymph node-metastasis (TNM) stage, Rab1A overexpression in the tumor tissues of a gastric cancer (GC) cohort was strongly correlated with poor prognosis in the patients. In addition, Rab1A knockdown significantly inhibited the *in vitro* proliferation and migration abilities of GC cells, as well as the growth of GC xenografts *in vivo*. Furthermore, a positive correlation was observed between Rab1A expression levels and that of different upstream/downstream mTOR targets. Taken together, Rab1A regulates the PI3K-AKT-mTORC1 pathway through the mTORC1 complex consisting of mTORC1, Rheb and Rab1A, and is a promising therapeutic target in GC.

## Introduction

Cancer is associated with high mortality and morbidity^1,2^, with the gastrointestinal malignancies accounting for a large proportion of the cases worldwide. Despite significant improvements in anti-cancer therapies over the past decades, the overall survival (OS) of patients is still poor^3^. The high morbidity, late diagnosis and availability of few targeted therapies are responsible for the high mortality rates seen in gastrointestinal cancers^4^, especially in China. Therefore, it is essential to identify the oncogenes involved in gastrointestinal cancer initiation and progression in order to develop biomarker-driven targeted therapy.

Rab proteins constitute one of the largest subfamilies of small GTPases, and are commonly regarded as housekeeping proteins that regulate intracellular membrane dynamics^5^. Rab1A is also a member of the Ras oncogene super family and plays an important role in vesicle transport^6^, along with mediating dynamic membrane trafficking from the endoplasmic reticulum (ER) to the Golgi^7,8^. Recent studies have shown that the Rab1A proteins are also involved in regulating signal transduction and autophagy^9,10^, and its aberrant expression levels are associated with proliferation, migration and drug-resistance in various cancers. Rab1A also activates mTORC1^5,11^, one of the two distinct kinases of the mTOR complex^12^, which controls cellular growth, metabolism and autophagy. Rapamycin, the mTORC1/2 target inhibitor, has shown promising therapeutic effects in various carcinomas^13,14^, although its overall objective response rate remains low. Rab1A exerts an influence on the PI3K-AKT-mTORC1 pathway via the mTORC1 complex^5^. However, little is known regarding the relationship between Rab1A and the upstream/downstream targets of mTORC1 in gastrointestinal cancers. Therefore, it is of biological and clinical significance to elucidate the effect of Rab1A in cancers, especially those of the gastrointestinal system.

In this study, we analyzed Rab1A expression levels in gastrointestinal cancer, as well as their correlation with clinico-pathological factors and the mTOR targets, using the GEPIA Platform. We also validated the role of Rab1A in gastric cancer (GC) patients, and its anti-cancer effects *in vitro* and *in vivo* by using the gene knockdown approach in order to identify a novel therapeutic target for GC.

## Results

### Expression levels of Rab1A in various kinds of cancer

**Figure 1:**
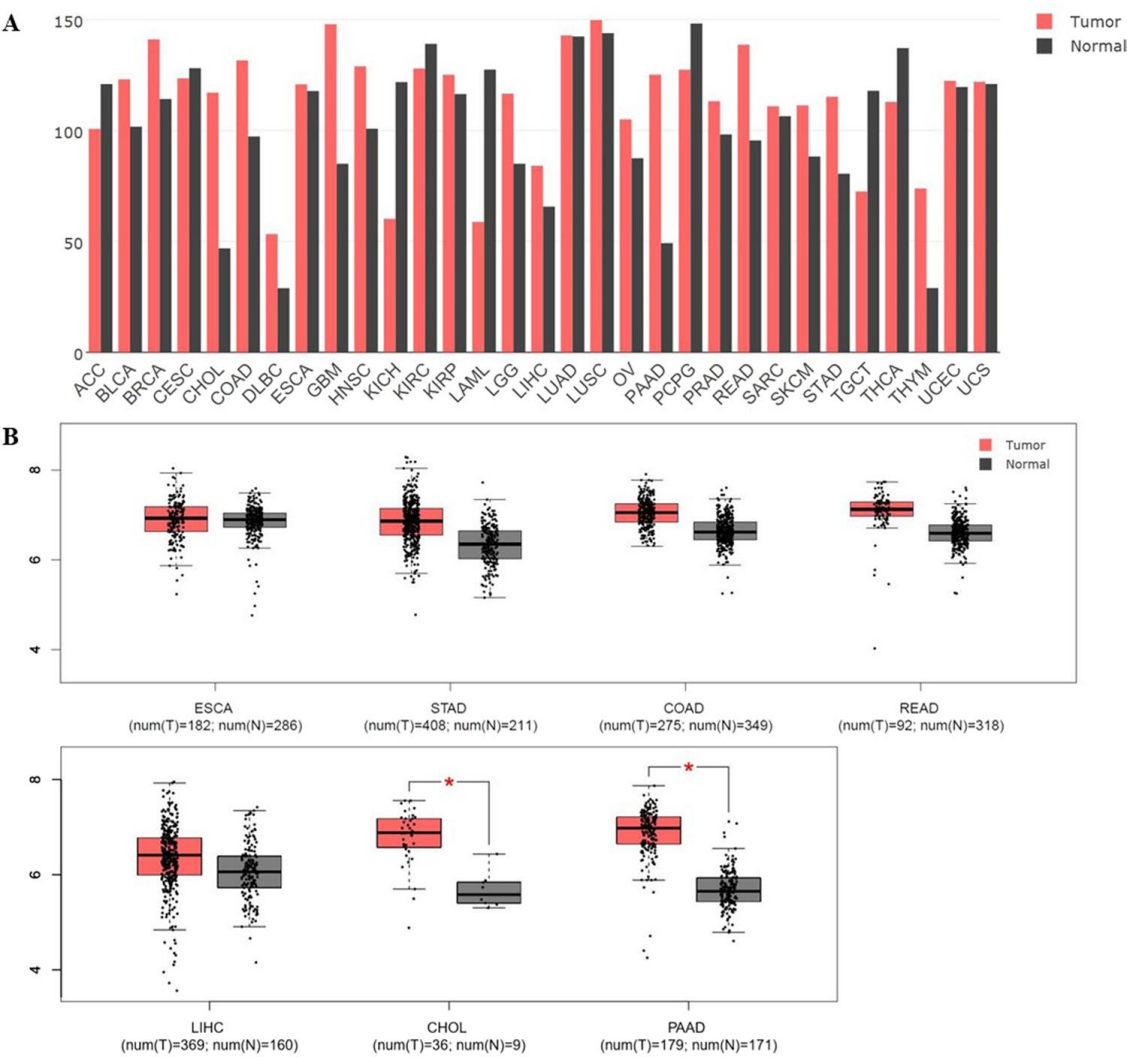
Expression of Rab1A in different cancer types in the GEPIA Platform. (**A**) Comparison of Rab1A levels in (**A**) multiple cancers, and (**B**) between the gastrointestinal tumor and paired normal tissues.

We analyzed the Rab1a expression levels in 31 cancer types via the GEPIA Platform (http://gepia.cancer-pku.cn), an online analysis website for The Cancer Genome Atlas (TCGA) and Gene Tissue Expression (GTEX) databases. Rab1A was significantly upregulated in the colorectal cancer (CRC) tissues compared to the para-tumor tissues, (Fig. 1A), and all the gastrointestinal cancers showed significantly higher expression levels of Rab1A compared to other cancers. We next analyzed the expression levels of Rab1A in these gastrointestinal cancers (Fig. 1B), and detected significantly higher levels in cholangiocarcinoma (CHOL) and pancreatic adenocarcinoma (PAAD) (P < 0.05), moderately higher levels in stomach adenocarcinoma (STAD), colon adenocarcinoma (COAD), rectum adenocarcinoma (READ) and liver hepatocellular carcinoma (LIHC), and similar levels in the esophageal carcinoma (ESCA) tumor tissues compared to their respective paired normal tissues.

### Expression levels of Rab1A in different TNM stages of gastrointestinal cancers

**Figure 2:**
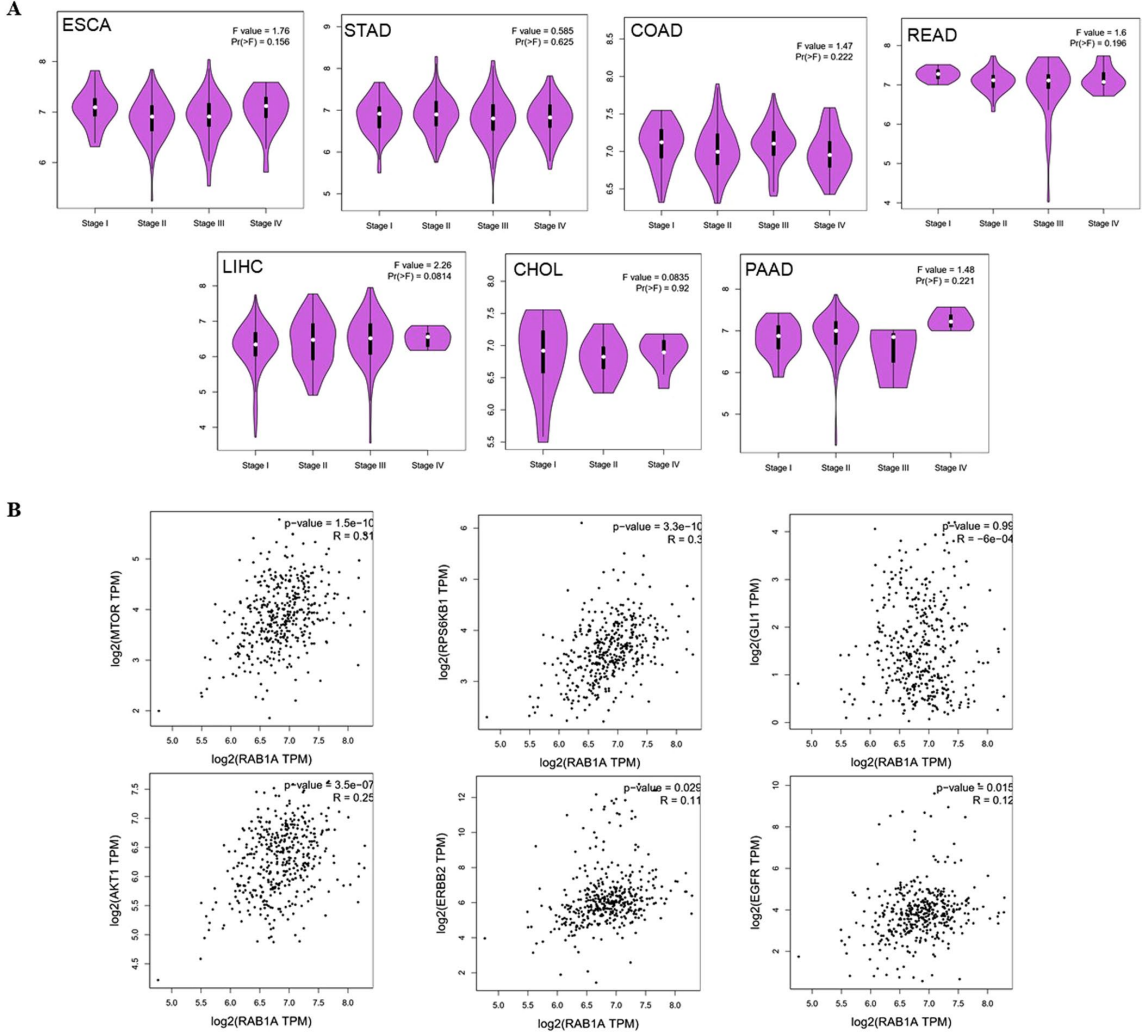
Rab1A expression correlated with TNM stage and mTOR targets in gastrointestinal cancers. (**A**) Rab1A expression in different TNM stages in esophageal carcinoma (ESCA), stomach adenocarcinoma (STAD), colon adenocarcinoma (COAD), rectum adenocarcinoma (READ), liver hepatocellular carcinoma (LIHC), cholangio carcinoma (CHOL) and pancreatic adenocarcinoma (PAAD). (**B**) Correlation analysis between Rab1A and upstream/downstream mTOR targets in the above cancers.

As described above, Rab1A expression levels were aberrantly high in the cholangio carcinoma, pancreatic adenocarcinoma, stomach adenocarcinoma, colon adenocarcinoma, rectum adenocarcinoma and liver hepatocellular carcinoma tissues, with cholangio carcinoma and pancreatic adenocarcinoma showing the most significant difference between the tumor and paired normal tissues. To further elucidate the relevance of Rab1A in gastrointestinal cancers, we analyzed its levels at the different TNM stages using the TCGA and GTEX databases, but found no obvious differences between early and advanced TNM stages (Fig. 2A). In sum, these results obtained from the TCGA and GTEX databases revealed that in various tumor stages (T stage), Rab1A expression in cholangio carcinoma, pancreatic adenocarcinoma, stomach adenocarcinoma, colon adenocarcinoma, rectum adenocarcinoma and liver hepatocellular carcinoma tissues was higher than paired controls. In contrast, compare the early and advanced gastrointestinal relevant cancer, Rab1A expression was not statistically different.

**Figure 3:**
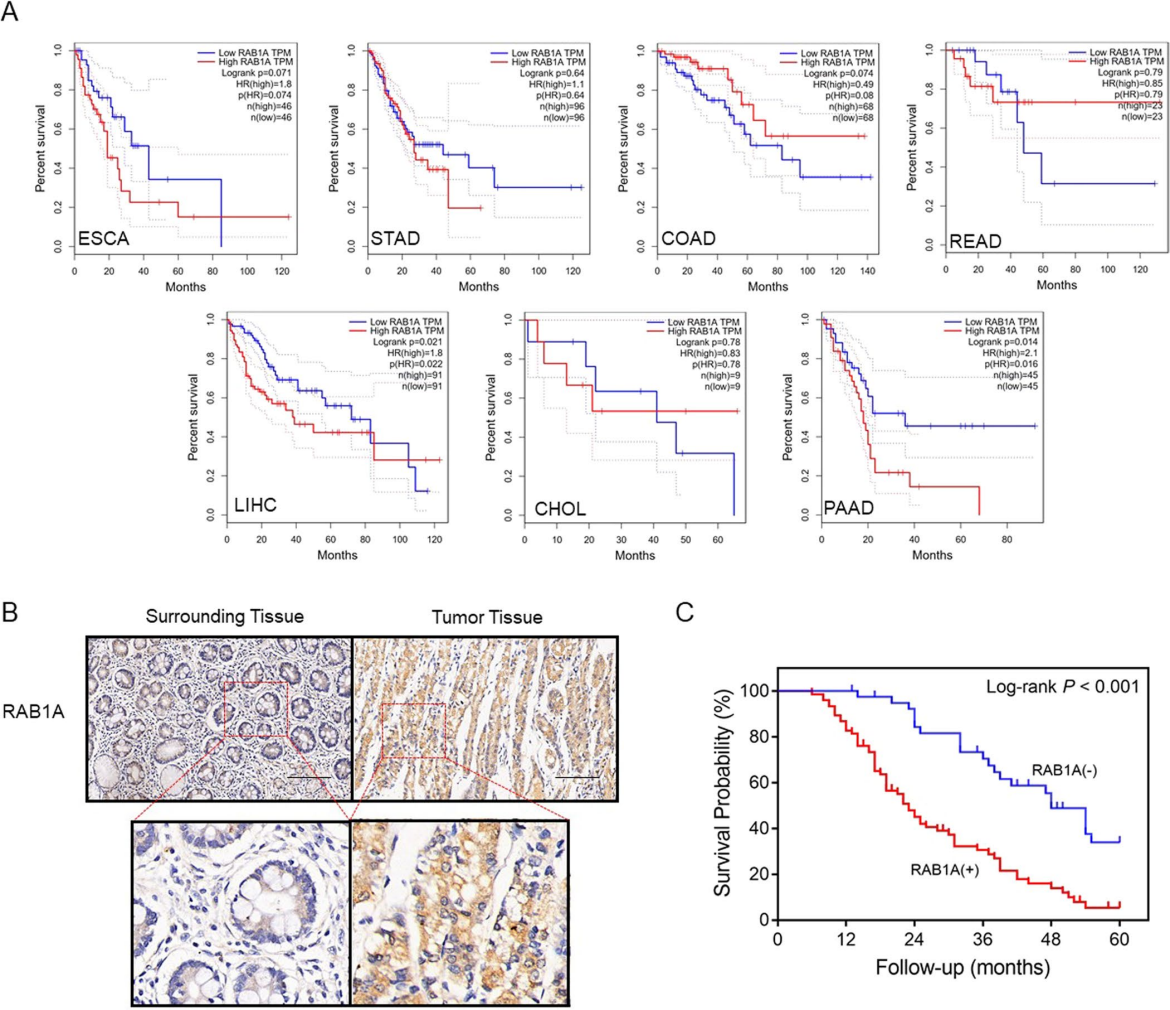
Prognostic relevance of Rab1A overexpression in gastrointestinal cancers. (**A**) The overall survival (OS) of esophageal carcinoma (ESCA), stomach adenocarcinoma (STAD), colon adenocarcinoma (COAD), rectum adenocarcinoma (READ), liver hepatocellular carcinoma (LIHC), cholangio carcinoma (CHOL) and pancreatic adenocarcinoma (PAAD) patients classified on the basis of Rab1A expression. (**B**) Rab1A expression in the paired tumor and healthy tissues in GC patients. (**C**) OS of Rab1A^hi^ and Rab1A^lo^ GC patients.

To determine any prognostic role of Rab1A in the gastrointestinal cancers, we next assessed the overall survival in cholangio carcinoma, pancreatic adenocarcinoma, stomach adenocarcinoma, colon adenocarcinoma, rectum adenocarcinoma and liver hepatocellular carcinoma patients demarcated on the basis of Rab1A expression. According to a recent study, approximately 70% of gastrointestinal tumors are positive for Rab1A^10^. Consistent with this, we classified the patients into the Rab1A negative and positive groups using the lower quartile of Rab1A expression level as the threshold. We found that lower Rab1A levels correlated with better survival and a favorable prognosis in liver hepatocellular carcinoma (P = 0.021) and pancreatic adenocarcinoma (P = 0.016), while high levels of Rab1A resulted in slightly poor overall survival in the esophageal carcinoma (P = 0.074) and colon adenocarcinoma (P = 0.080) patients (Fig. 3A).

### Correlation between Rab1A levels and mTOR targets in gastrointestinal cancers

Since high Rab1A levels attenuate survival, and Rab1A is an activator of mTORC1, we also analyzed the correlation between its expression status in all gastrointestinal cancers and the upstream (HER2, EGFR and AKT), downstream (S6K) and crosstalk (GLI1) targets of mTOR. Findings from TCGA and GTEX databases revealed no correlation between Rab1A and GLI1 levels in stomach adenocarcinoma, while that of HER2, EGFR, AKT, mTOR and S6K were significantly correlated with Rab1A (P < 0.05) (Fig. 2B). In addition, all 7 gastrointestinal cancers studied showed a significant association between Rab1a and mTOR expression levels (P < 0.05). Furthermore, Rab1A was associated with S6K levels in 7 cancers, with Gli1 in 2 cancers, AKT in 7 cancers, HER2 in 2 cancers, and with EGFR in 6 cancers (Table 1). Taken together, the factors upstream and downstream of mTOR are potential targets of Rab1A in gastrointestinal cancers, and therefore may have a therapeutic relevance.

**Table 1:** The relationship between the expressions of Rab1A and mTOR relevant gene targets including HER2, EGFR, AKT, S6K, GLI1 and mTOR in TCGA database.

	Rab1A							No. of
	ESCA	STAD	COAD	READ	LIHC	CHOL	PAAD	P < 0.05
mTOR	0.022	<0.001	<0.001	<0.001	<0.001	0.004	<0.001	7
S6K	<0.001	<0.001	<0.001	<0.001	<0.001	0.018	<0.001	7
GLI1	0.751	0.986	0.172	0.172	<0.001	0.573	<0.001	2
AKT	0.024	<0.001	<0.001	<0.001	<0.001	0.001	<0.001	7
HER2	0.847	0.029	0.284	0.284	0.001	0.550	0.211	2
EGFR	0.120	0.015	0.009	<0.001	<0.001	0.026	<0.001	6

Abbreviations: ACC: adrenocortical carcinoma. BLCA: bladder urothelial carcinoma. BRCA: breast invasive carcinoma. CESC: cervical squamous cell carcinoma and endocervical adenocarcinoma. CHOL: cholangio carcinoma. COAD: colon adenocarcinoma. DLBC: lymphoid neoplasm diffused large B-cell lymphoma. ESCA: esophageal carcinoma. GBM: glioblastoma multiforme. HNSC: head and neck squamous cell carcinoma. KICH: kidney chromophobe. KIRC: kidney renal clear cell carcinoma. KIRP: kidney renal papillary cell carcinoma. LAML: acute myeloid leukemia. LGG: brain lower grade glioma. LIHC: liver hepatocellular carcinoma. LUAD: lung adenocarcinoma. LUSC: lung squamous cell carcinoma. OV: ovarian serous cystadenocarcinoma. PAAD: pancreatic adenocarcinoma. PCPG: pheochromocytoma and paraganglioma. PRAD: prostate adenocarcinoma. READ: rectum adenocarcinoma. SARC: sarcoma. SKCM: skin cutaneous melanoma. STAD: stomach adenocarcinoma. TGCT: testicular germ cell tumors. THCA: thyroid carcinoma. THYM: thymoma. UCEC: uterine corpus endometrial carcinoma. UCS: uterine carcinosarcoma.

### Rab1A overexpression correlates with poor prognosis in GC patients

To further confirm any prognostic relevance of the aberrantly high levels of Rab1A in various gastrointestinal cancers, we analyzed the *in situ* Rab1A expression in paired tumor and adjacent normal tissues in GC patients. Rab1A expression was significantly higher in GC tissues compared to the para-cancer tissues in the entire patient cohort (P < 0.001) (Fig. 3B), and the OS of the Rab1A^hi^ group was considerably lower compared to that of the Rab1A^lo^ patients (P < 0.001) (Fig. 3C). Thus, Rab1a overexpression in GC is correlated to worse survival and prognosis of the patients, which is consistent with the results obtained from TCGA and GTEX databases.

### Rab1A expression correlates with that of mTOR targets in human GC cell lines

**Figure 4:**
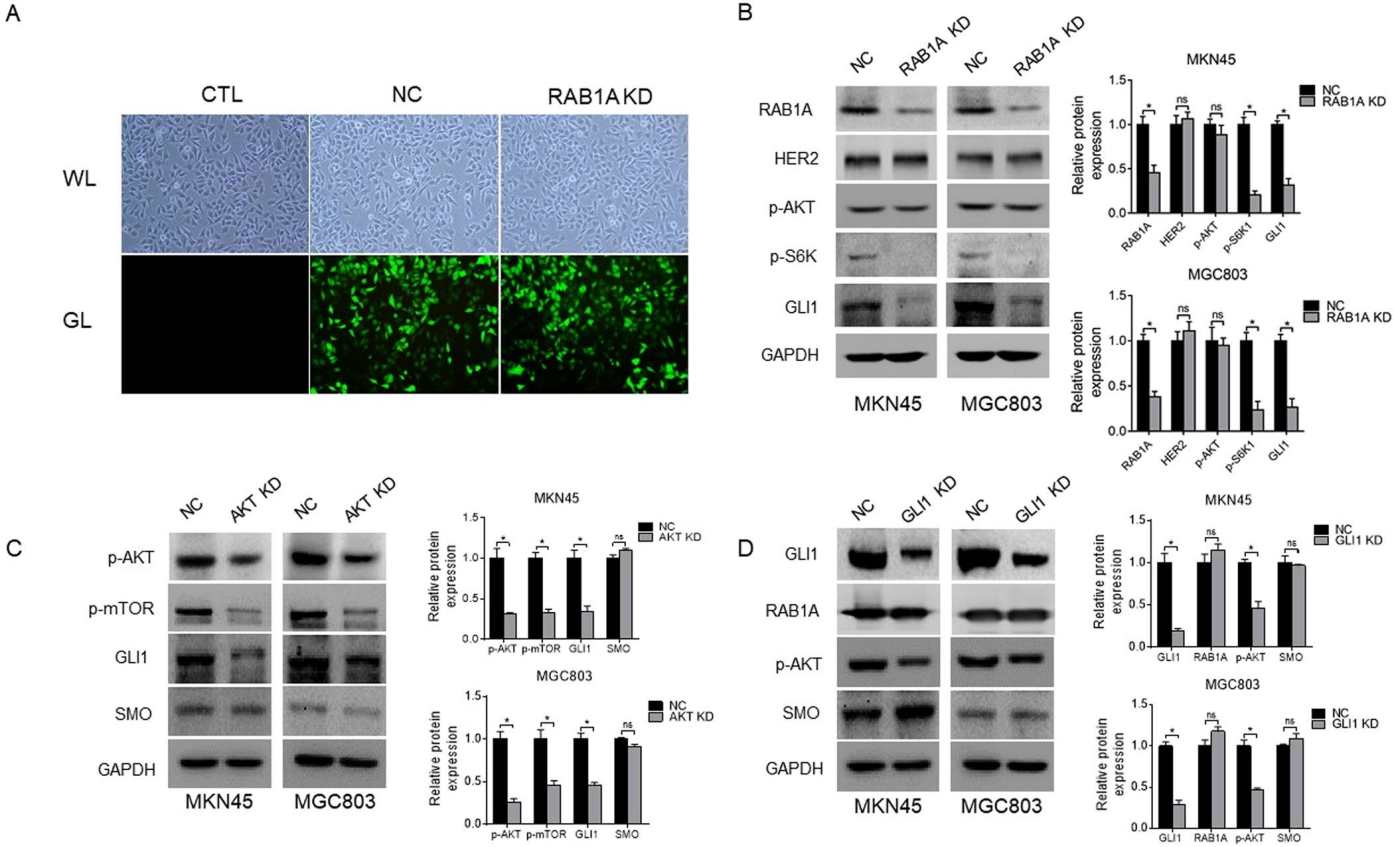
The relationship between Rab1A expression and the mTOR complex in GC cells. (**A**) Representative fluorescence imaging showing transfection efficiency in untreated control (CTL), shRab1A (Rab1A KD) and negative control shRNA (NC) groups. (**B**) Immunoblot showing levels of Rab1A, HER2, p-AKT, p-S6K and GLI1 protein in MKN45 cells and MGC803 cells transfected with shRab1A (Rab1A KD) or negative control shRNA (NC). (**C**) Immunoblot showing levels of p-AKT, p-mTOR, GLI1 and SMO protein in MKN45 cells and MGC803 cells after AKT knockdown. (**D**) Immunoblot showing levels of Gli1, Rab1A, p-AKT and SMO protein in MKN45 cells and MGC803 cells after GLI1 knockdown.

To further explore the relationship between mTOR targets and Rab1A in GC, we knocked down Rab1A in the MKN45 and MGC803 cells using the specific shRNA (Fig. 4A,B), and analyzed the expression levels of HER2, p-AKT, p-S6K and GLI1. Interestingly, the p-S6K and GLI1 levels decreased significantly after Rab1A knockdown (P < 0.05, Fig. 4B), while no significant changes were observed in HER2 and p-AKT expression (P > 0.05, Fig. 4B). Furthermore, knocking down AKT significantly downregulated p-mTOR and GLI1 (P < 0.05, Fig. 4C), but had no effect on the SMO levels (P > 0.05, Fig. 4C). To explore a possible feedback loop between p-AKT and GLI1, we knocked down the latter, and detected significant decrease in p-AKT levels but no significant changes in that of Rab1A and SMO (P > 0.05, Fig. 4D).

### Rab1A knockdown inhibits migration and proliferation of GC cells

**Figure 5:**
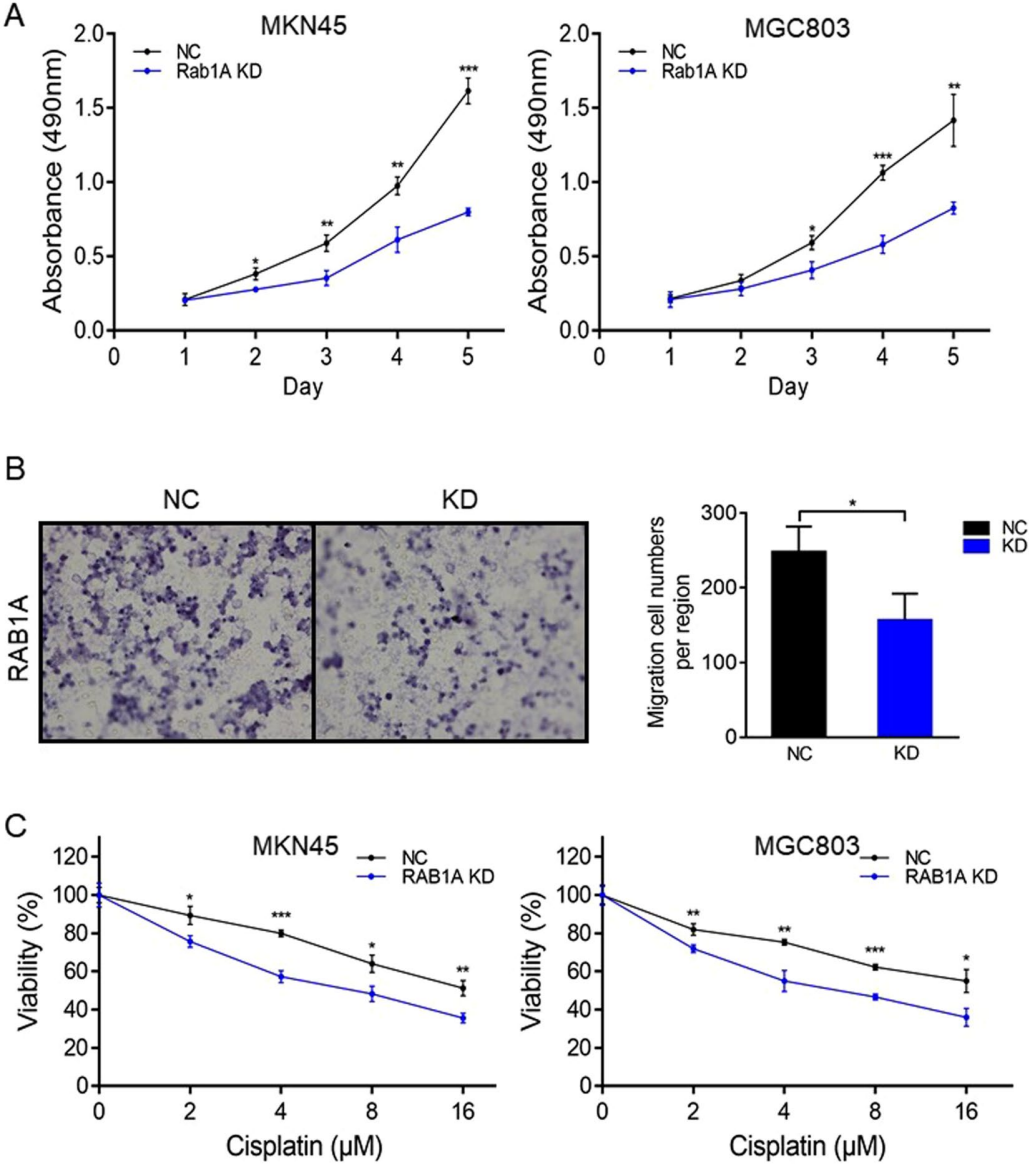
Effect of Rab1A on GC cell migration, proliferation and chemo-sensitivity. (**A**) MTT assay results showing the proliferation ability of MKN45 cells and MGC803 cells transfected with negative control shRNA (NC) or shRab1A (Rab1A KD). Data are presented as mean ± S.E.M. (n = 3). (**B**) Migration rates of MKN45 cells and MGC803 cells transfected with negative control shRNA (NC) or shRab1A (Rab1A KD). Representative photographs are of 200× magnification, and the relative number of migratory cells are presented as mean ± S.E.M. (n = 5). (**C**) Sensitivity analysis to Cisplatin. *P < 0.05, **P < 0.01, ***P < 0.001.

To determine the influence of Rab1A on the proliferation and migration ability of GC cell *in vitro*, we respectively performed the MTT and Transwell migration assays. The proliferative capacity of the Rab1A-knockdown MKN45/MGC803 cells was remarkably inhibited on days 1, 2, 3, 4 and 5 after 24 h of transfection (P < 0.05, Fig. 5A), as was the extent of migration compared to the cells transfected with control-shRNA (P < 0.05, Fig. 5B). Furthermore, the chemo-sensitivity of both cell lines treated with cisplatin for 48 hours increased significantly upon Rab1A knockdown (P < 0.05, Fig. 5C). Taken together, Rab1A is essential for the migration and proliferation of GC cells *in vitro*.

### Rab1A depletion inhibits GC progression *in vivo*

**Figure 6:**
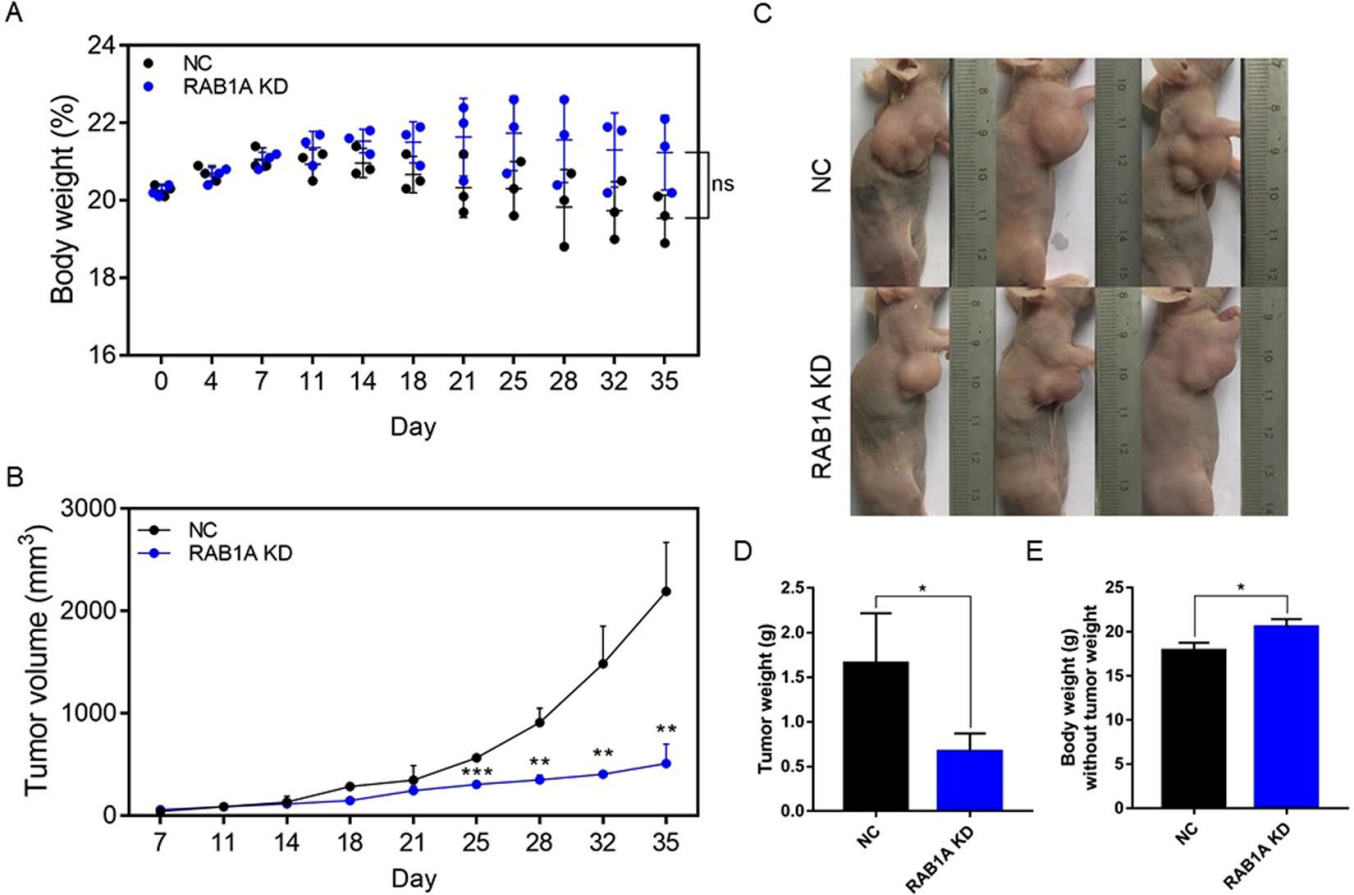
Rab1A knockdown suppresses GC tumor growth *in vivo*. (**A**) The body weight of the mice during the 5 week experiment. Data are presented as mean ± S.E.M. (**B**) The tumor volume during the indicated 5 weeks. Data are presented as mean ± S.E.M. (**C**) Representative pictures of mice inoculated with control and Rab1A-knockdown GC cells. (**D**) The weight of tumor masses after 5 weeks. Data are presented as mean ± S.E.M. (**E**) The body weight of the mice after subtracting the respective tumor weight. Data are presented as mean ± S.E.M.

To validate the *in vitro* neoplastic effects of Rab1A overexpression, we established GC xenografts in nude mice. Briefly, 5 × 10^6^ MKN45 GC cell transfected with control-shRNA or Rab1A-shRNA were implanted in the mice that were then monitored for 35 days. The mice treated with the Rab1A-knockdown GC cells weighed slightly more compared to those inoculated with the control GCs (P > 0.05, Fig. 6A). However, the Rab1A-knockdown tumors grew significantly slower compared to the control GC tumors, and showed less fluctuations in weight (P < 0.01, Fig. 6B,C). Interestingly, the Rab1A-knockdown tumors were also significantly lighter compared to the control (P < 0.05, Fig. 6D). Furthermore, it indicated the body weight without tumor weight treated with Rab1A depletion was significant heavier than the control group mice, which reached an obviously difference (P < 0.05, Fig. 6E). Taken together, Rab1A depletion retarded GC tumor growth in mice, indicating that it acts as an oncogene in GC progression.

## Discussion

Cancer is one of the most urgent and costly public health concerns worldwide, and causes more deaths compared to cardiovascular diseases. The gastrointestinal cancers in particular are highly prevalent and associated with high mortality rates^15^. Although the different therapeutic modalities against gastrointestinal cancers have advanced significantly, the prognosis of patients remains relatively poor due to distant metastasis and severe drug-resistance^16^. Thus, it is vital to identify novel therapeutic targets in order to improve the survival of the patients with gastrointestinal cancers.

Rab1A is a small GTPase that regulates ER-to-Golgi vesicular transport^17,18^. Recent studies show that Rab1A acts as an oncogene and is overexpressed in various cancers^19–21^, especially those affecting the gastrointestinal system, such as colorectal cancer^5^, gastric cancer^22^, hepatocellular carcinomas etc.^23,24^. Rab1A is an mTORC1 activator, through which it also regulates the PI3K-AKT-mTORC1 pathway. In the present study, we analyzed Rab1A expression levels in multiple gastrointestinal cancers using TCGA and GTEX databases. Compared to other cancers, the gastrointestinal types, especially CRC, showed significantly increased expression of Rab1A. Furthermore, Rab1A levels were significantly higher in cholangio carcinoma and pancreatic adenocarcinoma, and moderately higher in the stomach adenocarcinoma, colon adenocarcinoma, rectum adenocarcinoma and liver hepatocellular carcinoma tumors compared to their respective paired normal tissues. This strongly indicated that Rab1A likely acts as an oncogene in most, in not all, gastrointestinal cancers^25^.

Previous studies have reported that Rab1A overexpression is closely associated with tumor size and T stage^21^, which indicated a possible influence of Rab1A on the TNM stage as well. However, we did not observe any obvious changes in Rab1A expression between the early and advanced TNM stages in the above cancers. Recent studies have also correlated Rab1A overexpression with poor prognosis in several gastrointestinal cancers, such as CRC and GC^5,22^. Consistent with these reports, we observed that lower Rab1A expression indicated better prognosis in liver hepatocellular carcinoma and pancreatic adenocarcinoma. Furthermore, increased expression levels of Rab1A resulted in slightly poor overall survival in esophageal carcinoma and colon adenocarcinoma patients. To validate the database findings, we analyzed *in situ* Rab1A expression in the tumor and para-tumor tissues of GC patients, and not only observed aberrantly high levels of Rab1A in the former, but also a significant correlation between its overexpression and worse 5-year survival.

**Figure 7:**
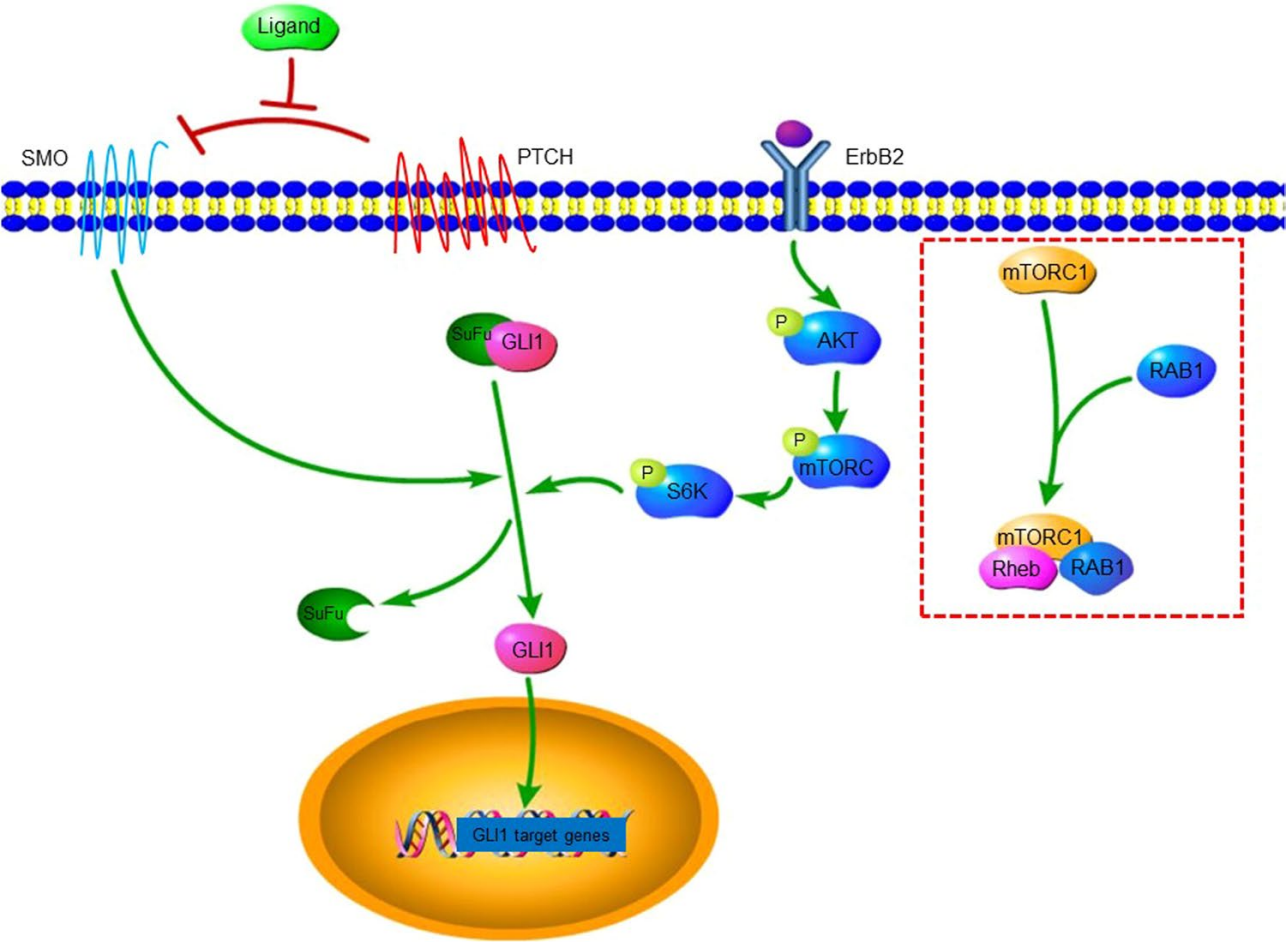
Rab1A regulates the PI3K-AKT-mTORC1 pathway through the mTORC1 complex consisting of mTORC1, Rheb and Rab1A.

S6K is the main downstream target of mTORC1, and a mechanistic link between Rab and p-S6K was first reported by Li *et al*. in 2010^26^. Recent studies have further established Rab1A as an activator of mTORC1 in CRC, prostate cancer and HCC^5,11,27^, and also shown a crosstalk between mTOR/S6K1 and Gli1^28–30^. To confirm the mechanism underlying Rab1A-mediated regulation of the AKT-mTORC1 pathway, we analyzed the correlation between the expression levels of Rab1A and the different mTOR targets. A significant association was observed between Rab1a and mTOR expression levels in all gastrointestinal cancers studied herein. In addition, Rab1a was correlated to S6K in 7 cancers, Gli1 in 2 cancers, AKT in 7 cancers, HER2 in 2 cancers, and EGFR in 6 cancers. These findings suggested that mTOR-related factors are also linked to Rab1A, and thus potential therapeutic targets in gastrointestinal cancers. Furthermore, p-S6K and GLI1 levels decreased significantly in human GC cell lines after knocking down Rab1A, while that of HER2 and p-AKT were unaffected. Furthermore, p-mTOR and GLI1 expression was also significantly reduced after AKT knockdown, while no significant change was observed in SMO expression levels. Finally, p-AKT and SMO levels were also unaffected by GLI1 knockdown. The putative regulatory circuit between Rab1A and the mTOR targets is illustrated in Fig. 7, which shows that Rab1A regulates the PI3K-AKT-mTORC1 pathway through the mTORC1 complex consisting of mTORC1, Rheb and Rab1A.

Recent reports indicate that Rab1A significantly promotes the growth and progression of several gastrointestinal cancers^5,11,31^, although a direct mechanistic role in GC has not been reported so far. To confirm the influence of Rab1A on GC, we knocked down its expression in two human GC cell lines. *In vitro* proliferation and migration abilities of the GC cells were significantly inhibited in the absence of Rab1A, and the xenografts of the Rab1A-knockdown GC cells also grew slower *in vivo*. These findings clearly showed that Rab1A is essential for GC tumor growth and metastasis, and that silencing Rab1A expression can result in a survival benefit. In conclusion, pharmacological inhibition of Rab1A is a promising targeted therapeutic strategy against GC.

## Conclusion

The novel finding of our study was that Rab1A is overexpressed in gastrointestinal cancers. Although not associated with the TNM stage, Rab1A overexpression significantly correlates with poor prognosis in GC patients. In addition, a positive correlation was observed between Rab1A expression levels and that of upstream/downstream mTOR targets, indicating that it influences the PI3K-AKT-mTORC1 pathway through the mTORC1 complex consisting of mTORC1, Rheb and Rab1A. Rab1A knockdown significantly abrogated GC cell growth *in vitro* and inhibited tumor progression *in vivo*, indicating a novel therapeutic target against gastrointestinal cancers.

## Methods

### Patients, tissue specimens and immuno-histochemical staining (IHC)

Paired tumor and normal tissues were obtained from 117 GC patients at the Department of General Surgery, Suzhou Municipal Hospital (Suzhou, China) from 2011–2013. Gastric adenocarcinoma was confirmed by histo-pathological examination. The study was approved by the Independent Ethics Committee (IEC) of Suzhou Municipal Hospital and all participants provided written informed consent. IHC was performed to determine *in situ* expression of Rab1A in the tissue samples. We declare that the method involving humans was performed in accordance with the relevant guidelines and regulations. And staining intensity was scored according to Yang *et al*.^32^.

### Short hairpin RNA transfection of human gastric cancer cell line

MKN45 and MGC803 lines were stably transfected with lentiviral constructs expressing Rab1A-specifc shRNA or scrambled control-shRNA. The shRNAs were synthesized by Gene Pharma (Shanghai, China). The cells were transfected with shRab1A or control-shRNA using Lipofectamine 2000 according to the manufacturer’s instructions, and selected with puromycin 72 hour later. Rab1A knockdown in the stable cell line was validated by fluorescence microscopy and Western blotting as described in our previous study^33^.

### Protein extraction and western blotting

Whole protein extracts from MKN45 and MGC803 were obtained by lysing the cells for 30 min in ice-cold RIPA buffer (Beyotime Inc., NanTong, China) as per the manufacturer’s protocol. Equal amount of protein per sample were separated by SDS-PAGE and transferred to nitrocellulose membranes. After blocking with 5% non-fat milk for 1 hour at room temperature, the blots were incubated overnight with the polyclonal primary antibodies, followed by the conjugated second antibodies for 1 hour at room temperature. The bands were developed by chemiluminescence and quantified by ImageJ software as previously described^34^.

### MTT assay

Cell viability was measured using an MTT assay kit (Amresco, USA) as per the manufacturer’s instructions. Briefly, the MTT solution was added to the cultured cells, and following incubation at 37 °C for 4 hours, the supernatants were removed and the formazan crystals were dissolved in 150 μl DMSO. The color was allowed to develop for ten minutes, and the absorbance was measured at 490 nm. The experiment was repeated three times, and the method has been described in our previous study^34^.

### Cell migration assay

The *in vitro* migration of the cells was tracked using a 24-well transwell plate (Corning Incorporated, USA). Briefly, the cells were seeded into the upper chambers in serum-free RPMI 1640, and the lower chamber was filled with complete RPMI 1640 supplemented with 10% FBS. After 24 h of culture, the cells that had migrated through the membrane were stained with 0·5% crystal violet and counted. The rate of migration was quantified by counting the number of cells in five random fields per sample as previously described^35^.

### Xenograft establishment

Male nude mice (BALB/c, SPF grade, weighing 16–18 g and 3–5 weeks old) were purchased from Shanghai SLRC laboratory Animal Co (Shanghai, China). The mice were subcutaneously injected with 5 × 10^6^ GC cells that were respectively transfected with control-shRNA or Rab1A-shRNA. The two groups nude mice were individually marked (n = 3 mice per group) after 3 days of feeding according to the weight and the day was recorded as 0.

The tumor bearing mice were sacrificed on day 35, and the tumors were resected for further analysis. We declare that the method involving mice was performed in accordance with the relevant guidelines and regulations. The animal experiments were approved by the Animal Ethics Committee of the Suzhou Municipal Hospital (Suzhou, China), and have been described in our previous study^33^.

### Statistical analysis

Data of all assays were expressed as the means ± S.E.M of three independent experiments. The overall survival was determined by the Kaplan–Meier method and compared by the log-rank test. The IHC results were analyzed by the Chi-squared test and the staining score means of two groups were compared by the *t*-test. P < 0.05 was considered statistically significant.

### Consent for publication

All the individuals provided written informed consent prior to enrolling in the study, and the study was approved by the Ethics Committees of Suzhou Municipal Hospital.

## Data Availability

Data are stored by the corresponding author of this paper and are available upon request.
